# “Clinicopathological profile of paragangliomas: A 5-Year retrospective analysis from a single tertiary Centre”

**DOI:** 10.1186/s13000-026-01771-1

**Published:** 2026-02-13

**Authors:** Geetha V, Megha Murali, Bhavna Nayal, Deepak Nayak, Vidya Monappa, Shreya Garg, P.S Priya, Girish Menon, Raghavendra Nayak, Kailesh Pujary, R. Balakrishnan, Suresh Pillai

**Affiliations:** 1 Department of Pathology, Kasturba Medical College, Manipal Academy of Higher Education, Manipal, Karnataka 576104 India; 2 Department of Radiodiagnosis, Kasturba Medical College, Manipal Academy of Higher Education, Manipal, Karnataka 576104 India; 3 Department of Neurosurgery, Kasturba Medical College, Manipal Academy of Higher Education, Manipal, Karnataka 576104 India; 4 Department of Otorhinolaryngology, Kasturba Medical College, Manipal Academy of Higher Education, Manipal, Karnataka 576104 India

**Keywords:** Paraganglioma, Neuroendocrine tumour, Metastasis, Immunohistochemistry, Clinicopathology

## Abstract

**Introduction:**

Paragangliomas are rare neuroendocrine tumours arising from neural crest–derived paraganglionic cells and form part of the pheochromocytoma–paraganglioma (PPGL) spectrum. The World Health Organization (WHO) 5th edition classification emphasizes autonomic origin, distinguishing sympathetic from parasympathetic paragangliomas, and recognizes that all paragangliomas possess metastatic potential.

**Objectives:**

To evaluate the clinicopathological features of paragangliomas diagnosed over a 5-year period at a single tertiary care centre.

**Methods:**

Twenty histologically confirmed cases of paraganglioma resected between 2018 and 2023 were retrospectively reviewed. Hematoxylin and eosin (H&E) and immunohistochemistry (IHC) slides were re-evaluated, and relevant clinical and radiological data were extracted from hospital records.

**Results:**

The cohort showed a female predominance (male-to-female ratio 1:1.8) with a median age of 49 years. The most common tumour site was the jugulotympanic region (30%), followed by intradural extramedullary (IDEM) locations (15%) and the retroperitoneum (15%). Two patients (10%) had metastatic disease involving the spine. Radiological findings were concordant with histology in 50% of cases. IHC was performed in 75% of cases and aided differentiation from histologic mimics. The Ki-67 proliferation index ranged from 1 to 2% in most cases, with one exception (4%). Follow-up data were available for 13 patients; one case showed local recurrence, and no syndromic associations were identified.

**Conclusion:**

Histological features alone are insufficient to predict tumour behaviour. Metastasis remains the only definitive criterion for malignancy, particularly when identified at sites lacking normal chromaffin tissue. Comprehensive clinicopathological assessment, incorporating radiology and immunohistochemistry, is essential for accurate diagnosis and management, and integration of molecular markers may further refine risk stratification in future studies.

**Supplementary Information:**

The online version contains supplementary material available at 10.1186/s13000-026-01771-1.

## Introduction

Paragangliomas are rare, non-epithelial neuroendocrine tumours arising from paraganglionic cells of neural crest origin. Together with pheochromocytomas, they constitute the spectrum of pheochromocytoma and paraganglioma (PPGL), as defined in the World Health Organization (WHO) 5th edition classification of Endocrine and Neuroendocrine Tumours. In contrast to earlier predominantly anatomic categorization, the current WHO classification emphasizes autonomic nervous system origin, formally distinguishing sympathetic from parasympathetic paragangliomas. Sympathetic paragangliomas, including adrenal pheochromocytomas, typically arise along the thoracolumbar sympathetic chain and are often catecholamine-secreting, whereas parasympathetic paragangliomas predominantly occur in the head and neck region and are usually non-functional [1].

In keeping with this updated framework, the traditional dichotomy of “benign” versus “malignant” paragangliomas is no longer recommended. All paragangliomas are regarded as tumours with metastatic potential, with metastatic spread to sites lacking normal paraganglionic tissue—such as bone, liver, lung, or lymph nodes—remaining the only definitive criterion of malignancy. The WHO 5th edition further emphasizes risk stratification based on clinical, anatomic, and molecular parameters rather than reliance on histomorphology alone, which has limited utility in predicting biological behaviour [1]. Given their rarity, wide anatomic distribution, and variable clinical course, evaluation of paragangliomas requires an integrated clinicopathological approach incorporating clinical presentation, radiological findings, histomorphology, and immunohistochemistry, with increasing emphasis on molecular and genetic context. Despite advances in classification and understanding of tumour biology, published data on the clinicopathological spectrum of paragangliomas—particularly from non-adrenal and extra-head-and-neck sites—remain limited and are often constrained by small sample sizes and geographic variability. Institutional series therefore continue to play an important role in elucidating diagnostic challenges, anatomic distribution, and patterns of clinical behaviour.

### Objective

To evaluate the anatomical distribution, histomorphology, immunoprofile, and clinical outcomes of paragangliomas, including metastatic behaviour and syndromic association.

### Methods

Twenty histologically confirmed cases of paraganglioma resected between 2018 and 2023 were retrospectively reviewed. Hematoxylin and eosin (H&E) and immunohistochemistry (IHC) slides were re-evaluated, and relevant clinical and radiological data were extracted from hospital records.

## Results

The male-to-female ratio was 1:1.8, with a median age of 49 years (range: 25–71 years). The most common tumour site was the jugulotympanic region (30%), followed by intradural extramedullary (IDEM) locations (15%) and the retroperitoneum (15%). Other anatomical sites are detailed in Table 1.

**Table 1:** Various sites of paraganglioma

Site	No of cases (*n* = 20)
1. Jugulotympanic paraganglia	6 (30%)
2. Intra-Dural extra medullary (IDEM)	3 (15%)
3. Retroperitoneum	3 (15%)
4. Carotid body	2 (10%)
5. Cerebello-pontine angle (CP angle)	1 (5%)
6. Urinary bladder	1 (5%)
7. Parotid	1 (5%)
8. External auditory canal	1 (5%)
9. Bone metastasis	2 (10%)

Radiological imaging was available in 14 of 20 cases and correlated with histology in 50% (7/14). Radiological differentials included schwannoma and myxopapillary ependymoma for IDEM lesions, gastrointestinal stromal tumour for retroperitoneal masses, and metastasis or carcinoma in cases involving the liver and urinary bladder. Two metastatic paragangliomas were initially interpreted radiologically as metastases of unknown primary.

**Fig. 1:**
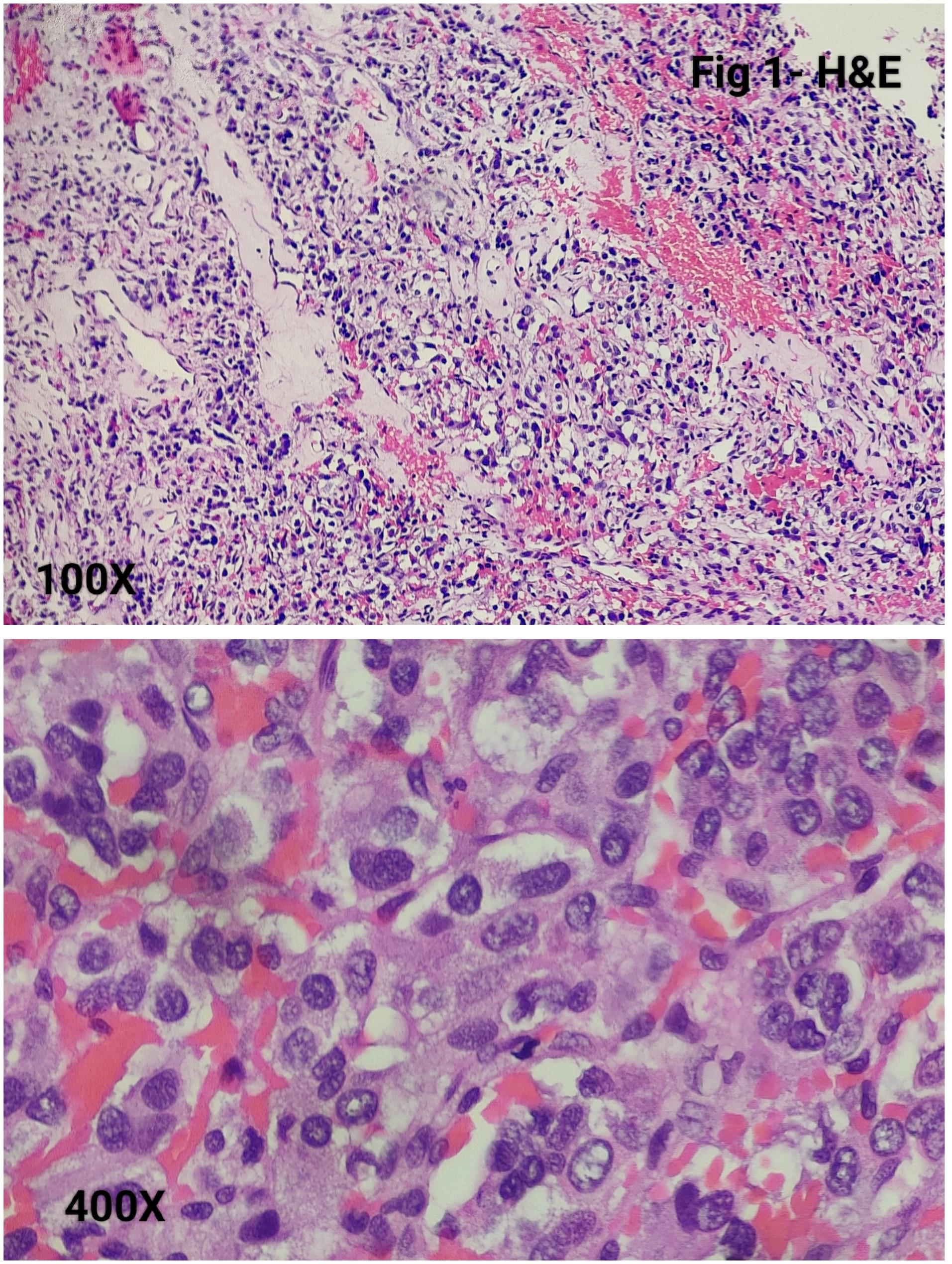
Photomicrograph shows tumor cells arranged in Zellballen pattern with individual cells showing stippled chromatin, occasional small nucleoli and basophilic to clear cytoplasm

**Fig. 2:**
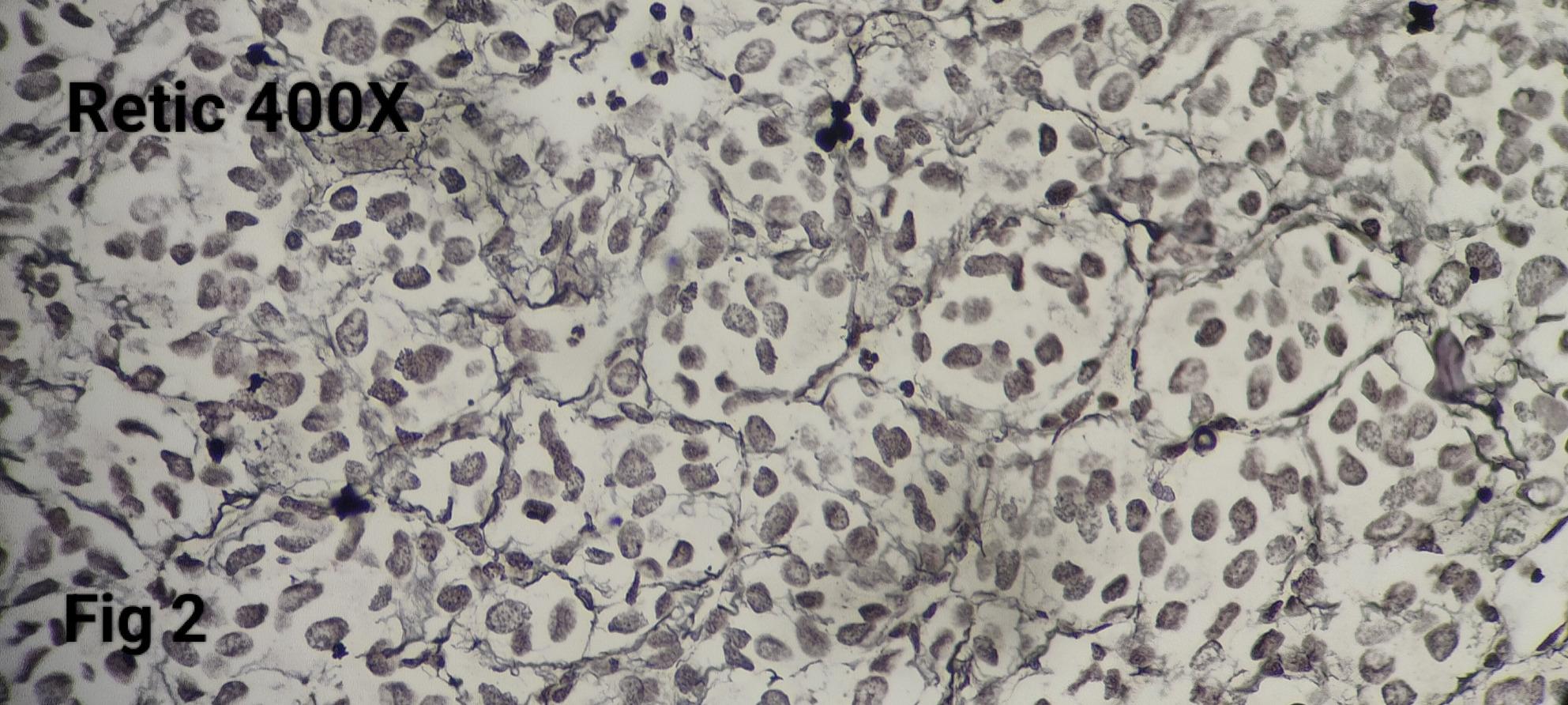
The Reticulin stain highlights the nested pattern

Morphologically, all tumours demonstrated a classic Zellballen architecture, with nests of chief cells surrounded by sustentacular cells (Fig. 1). The nested pattern was highlighted by reticulin staining (Fig. 2).

**Fig. 3:**
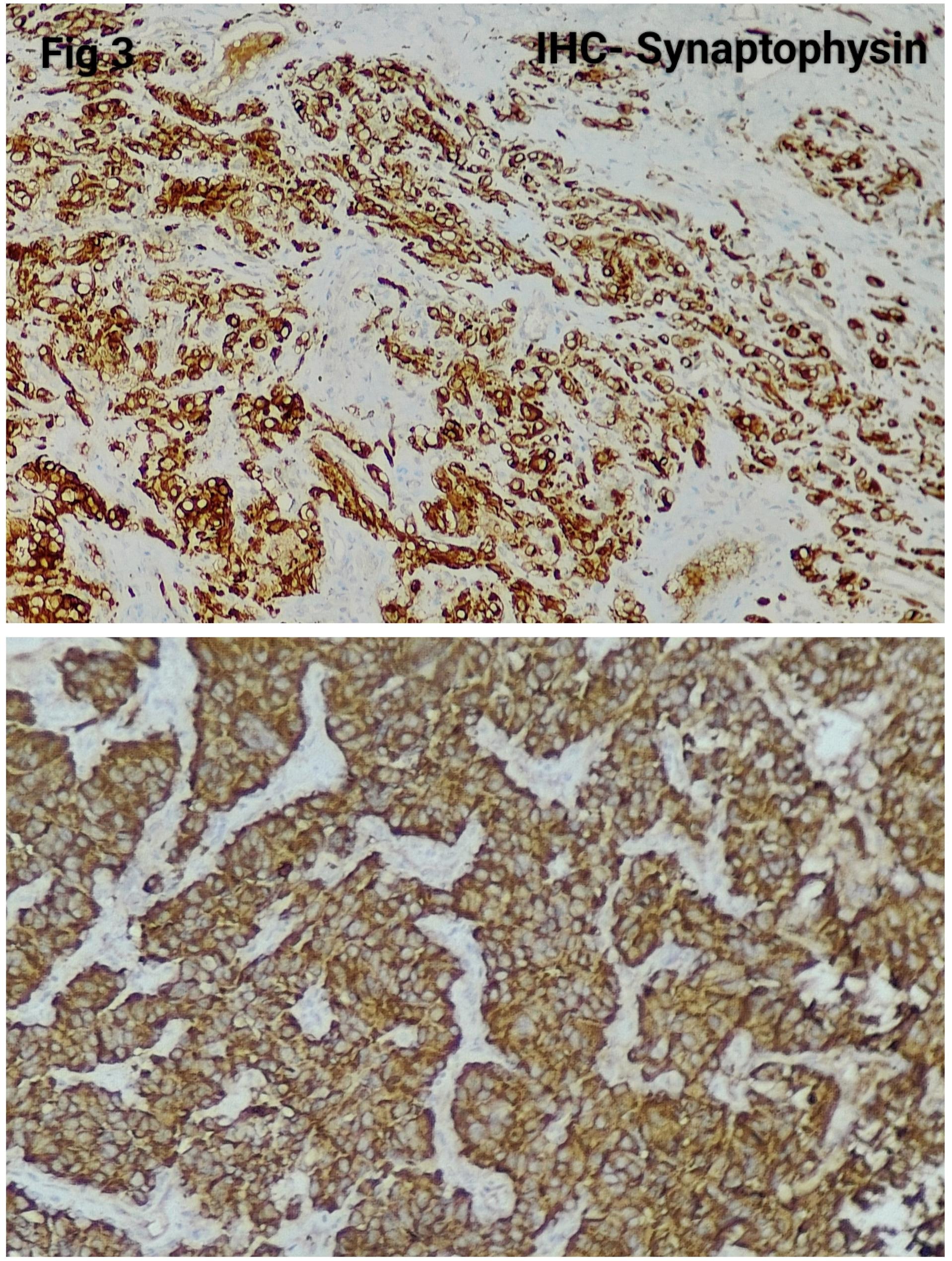
The tumor cells are Synaptophysin- positive

**Fig. 4:**
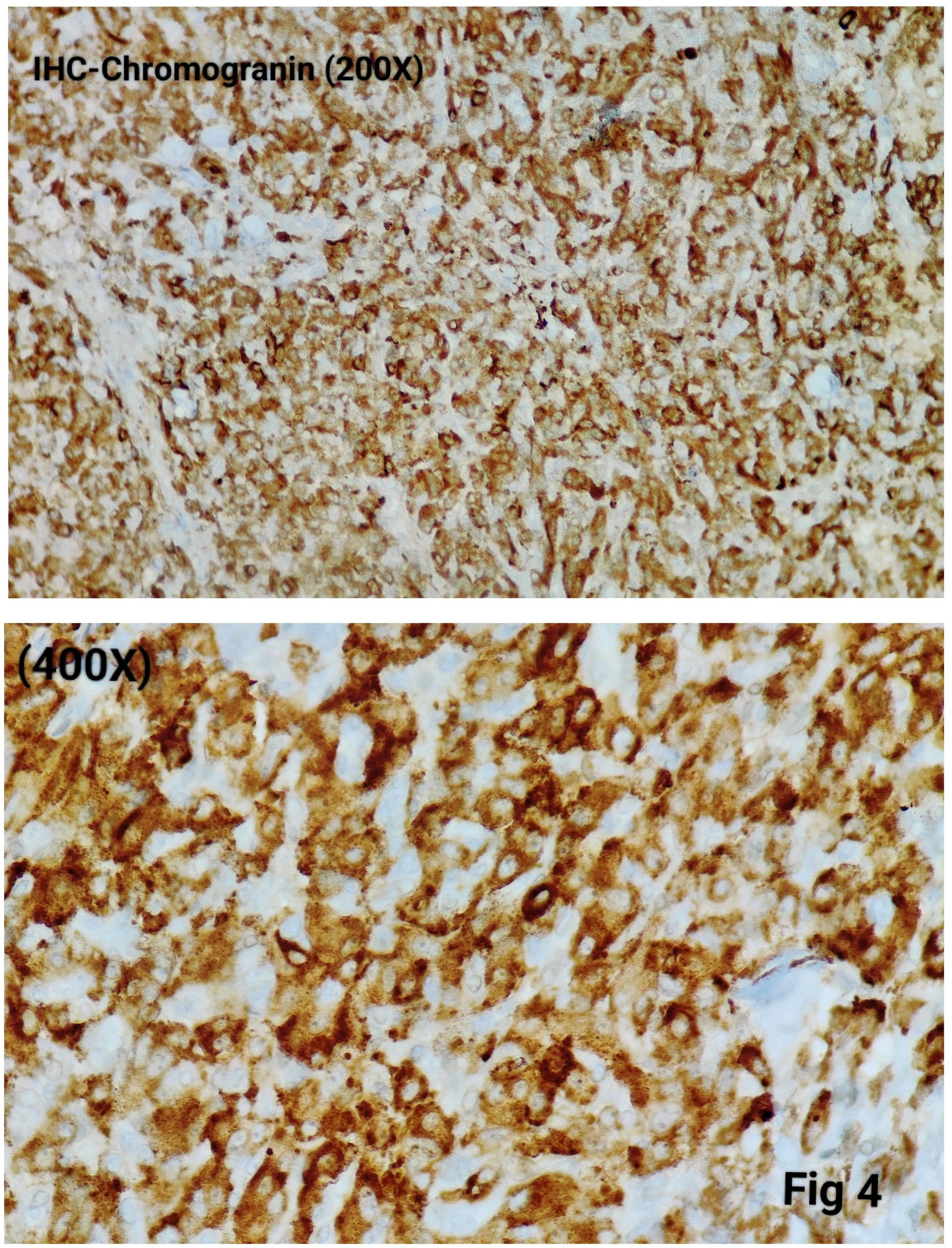
The tumor cells are Chromogranin- positive

**Fig. 5:**
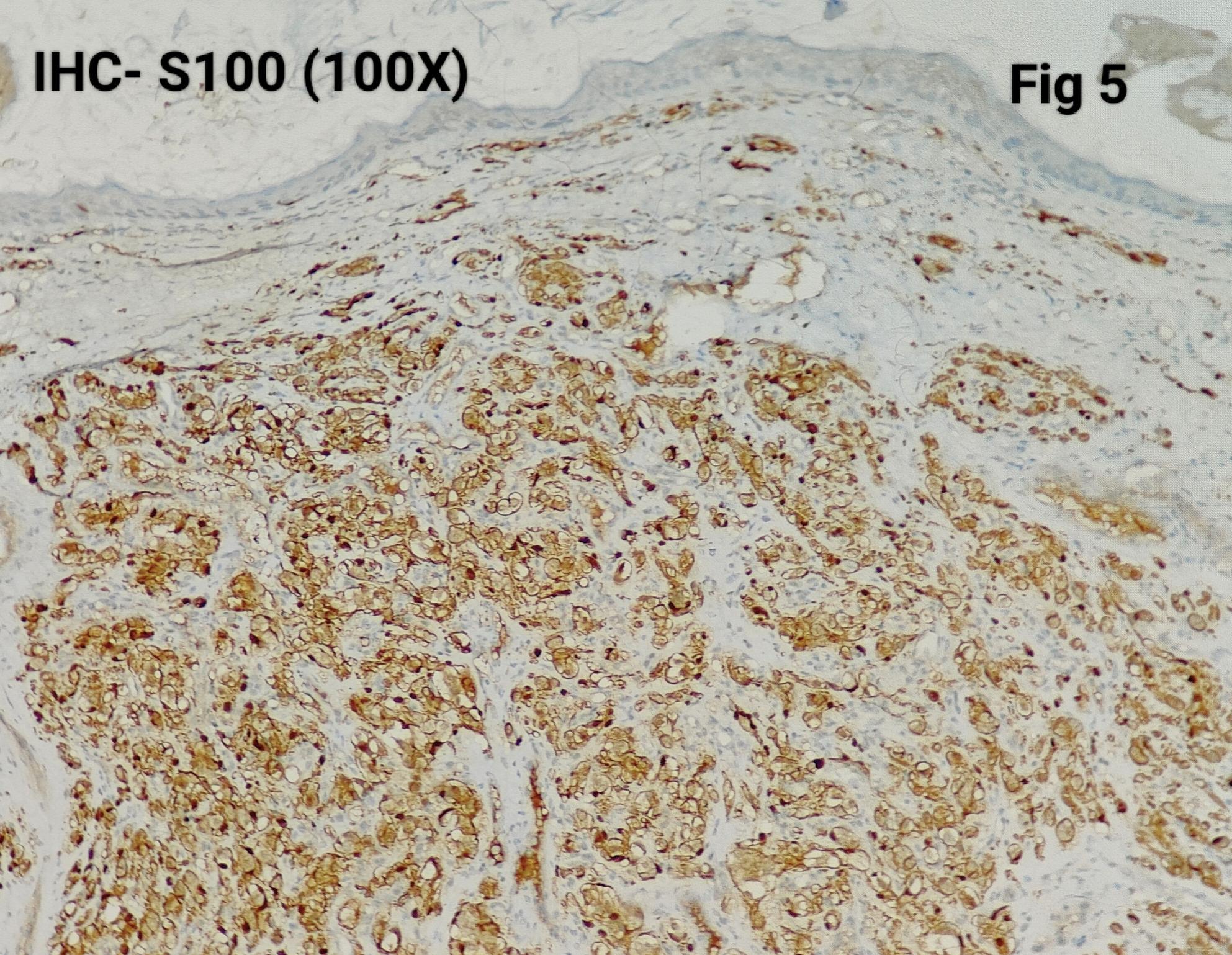
S100 positive in Sustentacular cells

**Fig. 6:**
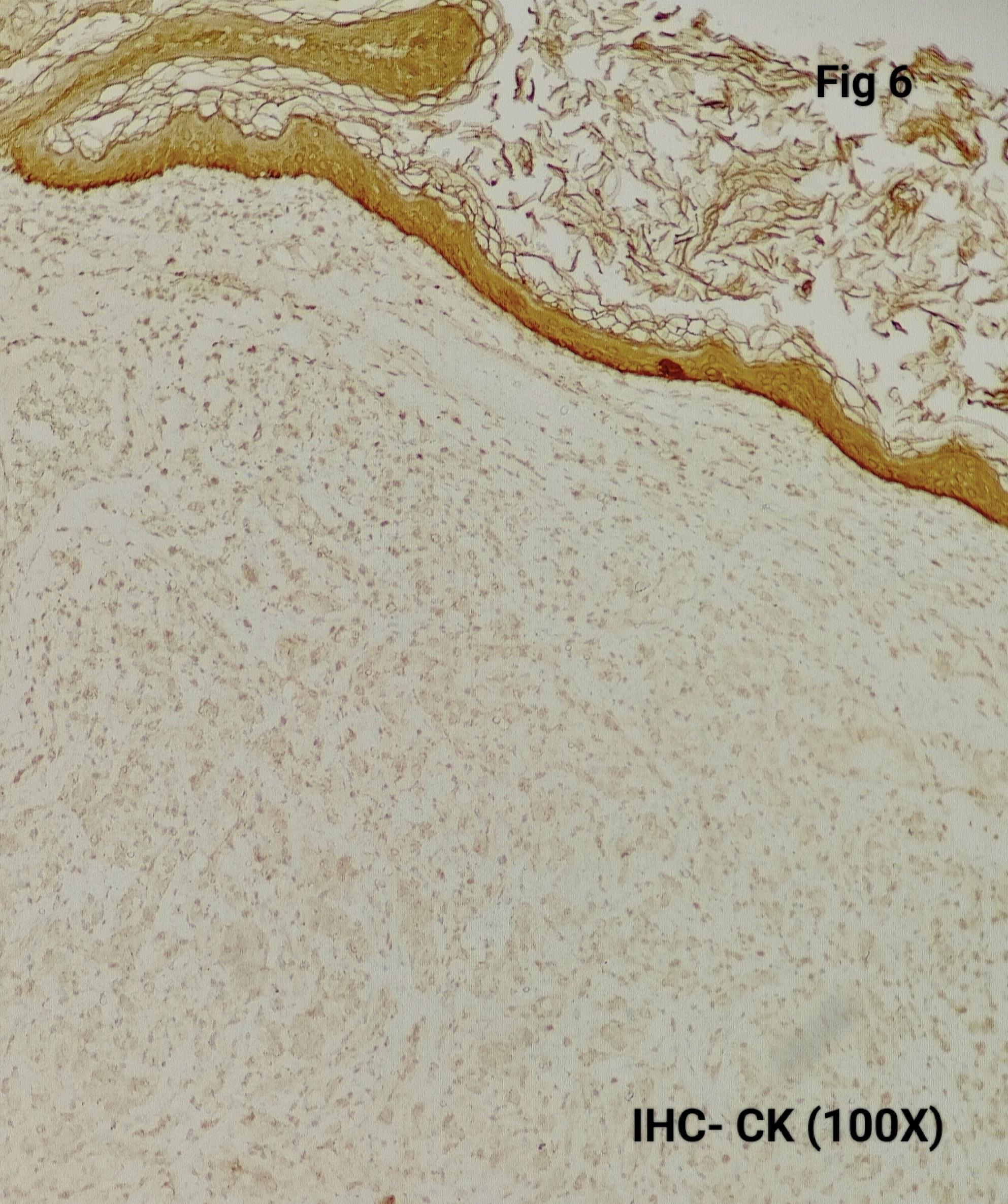
The tumor cells are CK Negative

**Fig. 7:**
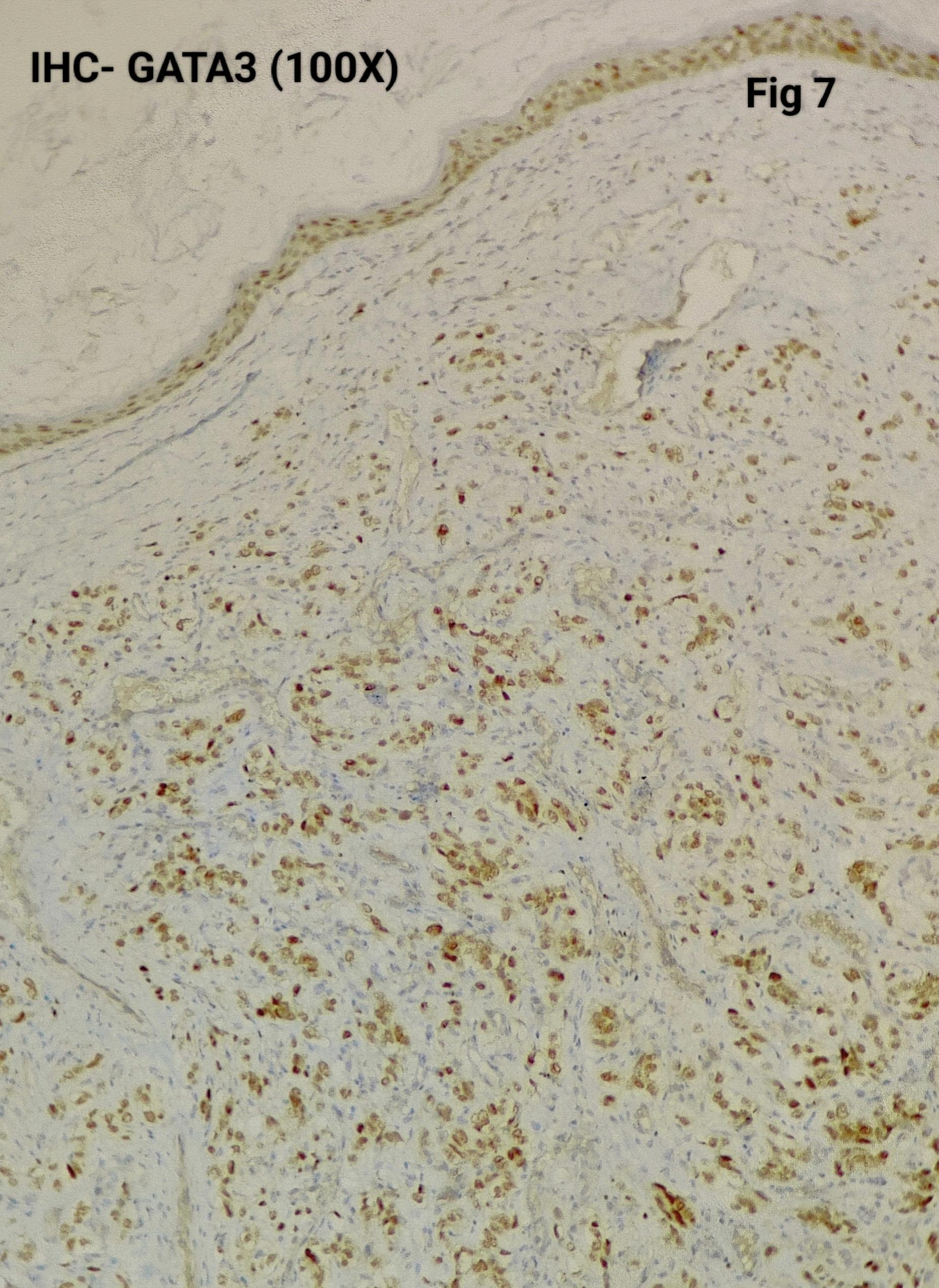
Shows GATA3 nuclear positivity

**Fig. 8:**
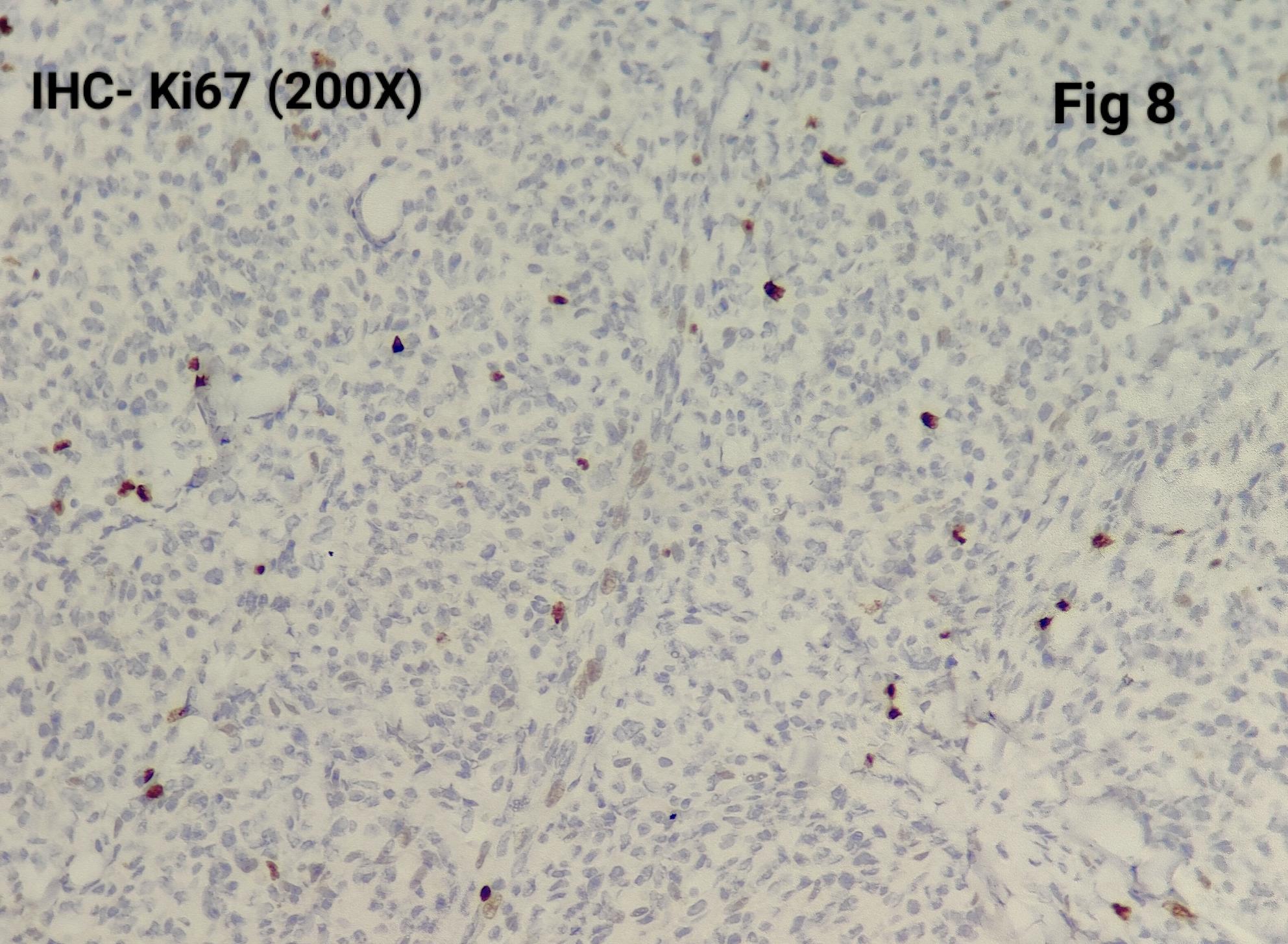
Ki67 around 2%

Immunohistochemistry was performed in 15 cases (75%) to confirm the diagnosis and exclude close differentials, particularly in metastatic settings. Tumour cells were positive for synaptophysin (Fig. 3), chromogranin (Fig. 4), S100 highlighted sustentacular cells (Fig. 5), while Cytokeratin was consistently negative (Fig. 6), aiding exclusion of epithelial neoplasms such as medullary thyroid carcinoma. Nuclear positivity for GATA3 is seen in Fig. 7. HMB-45 was performed in one case to exclude melanoma, while TTF-1 and PAX8 were performed in a retroperitoneal case evaluated initially as metastasis of unknown origin. The Ki-67 proliferation index ranged from 1 to 2% (Fig. 8) in most cases, with a single exception showing approximately 4%. The key morphological and immunohistochemical features distinguishing paraganglioma from its close differentials, including medullary thyroid carcinoma and metastatic melanoma, are summarized in Table 2.

**Table 2:** Summarises the morphological and immunohistochemical difference between Paraganglioma, medullary thyroid carcinoma and metastatic melanoma [8–10]

Feature	Paraganglioma	Medullary Thyroid Carcinoma	Metastatic Melanoma
**Architecture**	Zellballen nests with rich vasculature	Sheets, trabeculae, or nests	Discohesive nests, prominent nucleoli
**Chief cells**	Chromogranin+, Synaptophysin+, NSE+, **Cytokeratin–**, Calcitonin–	Calcitonin+, CEA+, Cytokeratin+	S-100+, HMB-45/Melan-A+, **Melanocytic markers**
**Sustentacular cells**	S-100 + around nests	Absent or rare	Not applicable

Follow-up data were available for 13 patients. One patient developed local recurrence at the site of a glomus tympanum tumour after one year. Two patients, aged 26 and 35 years, developed metastatic disease involving the D9 and D1–D2 vertebrae, respectively, and received CVD (Cyclophosphamide, Vincristine, and Dacarbazine) chemotherapy. Genetic counselling and testing were performed in both cases. No syndromic associations were identified. The most common postoperative complication in jugulotympanic tumours was facial nerve palsy (3/6), followed by hypoglossal nerve palsy (1/6).

## Discussion

Paragangliomas in our series demonstrated marked heterogeneity in anatomic distribution and clinical outcome, reflecting the biological unpredictability of these tumours. Consistent with WHO recommendations, histomorphology alone was insufficient to stratify tumour behaviour, and metastatic spread to non-paraganglionic sites remained the only unequivocal indicator of aggressive disease [1].

Our cohort showed a female predominance, consistent with WHO data and findings reported by Smith et al. [2] but contrasting with the male predominance described by Feng et al. [3] and Mishra et al. [4]. The median age of 49 years was comparable to that reported by Mishra et al. [4]., while both metastatic cases in our study occurred in younger patients, supporting observations that aggressive disease may present earlier in life [1].

The jugulotympanic region was the most common site in our cohort, differing from Feng et al. [3], who reported carotid body predominance, and from Mishra et al. [4], who focused on spinal paragangliomas. Rare primary sites identified in our study—including the cerebellopontine angle, urinary bladder, parotid gland, and external auditory canal—underscore the anatomical diversity of these tumours.

Radiological misclassification occurred in nearly half of cases, highlighting the diagnostic challenge posed by paragangliomas at unusual sites. Immunohistochemistry therefore plays a pivotal role in diagnosis and exclusion of histologic mimics. In accordance with WHO 5th edition recommendations, a core panel comprising synaptophysin, chromogranin, nuclear GATA3 for chief cells, and S100 for identification of sustentacular cells was employed, with cytokeratin negativity aiding exclusion of epithelial neoplasms. Adjunct markers such as TTF-1, PAX8, and HMB-45 were applied selectively in challenging cases. Although Ki-67 was routinely assessed, its low values and lack of correlation with outcome reinforced its limited prognostic utility.

While the WHO 5th edition emphasizes SDHB immunohistochemistry as a screening tool for SDHx-related disease, SDHB staining was not routinely performed in the present study due to non-availability. Nevertheless, recognition of established clinicopathological risk factors remains valuable in identifying patients who may benefit from targeted genetic evaluation and closer follow-up.

One patient experienced local recurrence, and two developed metastatic disease, rates consistent with published estimates for extra-adrenal paragangliomas [5]. Unlike Feng et al., [3] who reported lymph nodes as the most common metastatic site, bone was the predominant site in our series. Neither morphology nor Ki-67 reliably predicted aggressive behaviour. A substantial proportion of paragangliomas are associated with hereditary syndromes, most commonly involving SDHx gene mutations, but also occurring in the context of multiple endocrine neoplasia type 2, von Hippel–Lindau disease, and neurofibromatosis type 1, underscoring the importance of genetic counseling and evaluation [1, 6, 7]. No syndromic association was identified in the present cohort, although limited access to routine genetic testing may have contributed to under-recognition.

Mishra et al. [4] highlighted the frequent radiological misdiagnosis of spinal paragangliomas as ependymomas, a finding corroborated in our IDEM cases. Smith et al. demonstrated that head and neck paragangliomas are largely indolent tumours with low recurrence and metastatic rates, supporting a conservative, risk-adapted management approach [2]. Comparative analysis with prior studies is summarized in Table 3.

**Table 3:** Compares the present study with previously published series

Sample size/ years	Our study	WHO^1^	Feng *N* et al.^3^	Misha et al.^4^ (spinal paragangliomas)	Smith et al. ^2^ (HNPGLs)
	20/ 5 years	Not applicable	152/32 years	8/6 years	194/20 years
1. Median age	49 years	41–47 years (mean age)	43 years	50.4 years	50 years
2. Male: female ratio	1:1.8 (female predominance)	1:1.7 (female predominance)	1.3:1 (male predominance)	3:1 (male predominance)	1:1.7 (female predominance)
3. Most common site	Jugulotympanic paraganglia	Carotid body	Retroperitoneum	Not applicable	Carotid body
4. Spine	IDEM Clinical diagnosis was schwannoma (2/3) and myxopapillary ependymoma (1/8)		Not applicable	IDEM in all cases Clinical diagnosis was schwannoma (5/8) and ependymoma (3/8)	Not applicable
5. Most uncommon site	1. Cerebello-pontine angle 2. Urinary bladder 3. Parotid 4. External auditory canal	Larynx	1. Mesothelium 2. Lung		Vagal / tympanic
6. No of cases of metastasis with site	2/20 Bone	Most common site is lymph node followed by bone, lung and liver	10/152 Lymph node	NIL	Uncommon
7. Recurrence	1/20	Not applicable	1/152	NIL	Low (not specified)

Recent literature increasingly emphasizes integrated risk stratification models incorporating tumour site, SDHx mutation status, size, and biochemical profile rather than morphology alone [6, 11]. Molecular classification has identified distinct PPGL subgroups with differing biological behaviour, and SDHB mutations remain independently associated with metastatic risk [7, 12, 13]. Loss of ATRX expression, linked to alternative lengthening of telomeres, has also been associated with aggressive behaviour and may represent an emerging prognostic marker complementing SDHB status [14, 15]. Paragangliomas associated with VHL and SDHx mutations belong to the hypoxia signaling cluster, characterized by activation of hypoxia-inducible pathways and upregulation of downstream targets such as carbonic anhydrase IX (CA IX), reflecting pseudohypoxic tumor biology [6, 7, 13].

These emerging molecular pathways further emphasize that paragangliomas represent a biologically diverse group of tumors. Future studies incorporating comprehensive molecular profiling alongside traditional clinicopathological assessment are likely to improve prognostication and inform individualized patient management.

### Limitations

Limitations of this study include its retrospective design, limited sample size, and lack of routine molecular and genetic evaluation, including SDHB immunohistochemistry, due to institutional non-availability. These factors underscore the need for larger prospective studies integrating molecular data to refine prognostication.

## Conclusion

Paragangliomas exhibit a broad spectrum of clinicopathological features, with diagnosis and prognostication remaining challenging due to their variable presentation and limited predictive value of histology. In this 5-year series, the jugulotympanic region was the most frequently involved site, with a female predominance. Metastatic disease occurred in a minority of patients and could not be predicted by morphology or proliferation index alone.

Metastasis remains the only reliable indicator of malignant behaviour, particularly when identified in non-chromaffin tissues. Accurate diagnosis requires integration of clinical, radiological, and immunohistochemical findings to exclude histologic mimics. Genetic evaluation, particularly assessment of SDHx alterations and ATRX loss, should be considered in metastatic, multifocal, or early-onset cases to enhance risk stratification and long-term management. Multicentric studies incorporating standardized molecular testing will be essential to validate emerging prognostic markers across diverse populations.

## Supplementary Information

Supplementary Material 1.

## Data Availability

No datasets were generated or analysed during the current study.
