# Supplementary material for: “Clinicopathological profile of paragangliomas: A 5-Year retrospective analysis from a single tertiary Centre”
Source: Diagn Pathol. 2026 Feb 13;21:31. doi: 10.1186/s13000-026-01771-1 (PMC13005369; doi:10.1186/s13000-026-01771-1)
Supplement: Supplementary file 1 — Supplementary Material 1. [file 13000_2026_1771_MOESM1_ESM.docx]

1. **Paragangliomas can mimic other tumors** clinically and radiologically—accurate diagnosis requires histopathology and immunohistochemistry.
2. **Histological features and Ki-67 index are not predictive** of malignancy; only the presence of metastasis confirms malignancy.
3. **Bone was the exclusive site of metastasis** in this series, highlighting an atypical metastatic pattern.
4. **Sustentacular (S100+) and chief cell markers (Chromogranin, Synaptophysin, GATA3)** are crucial for confirmation and for excluding mimics like medullary carcinoma and melanoma.
5. **Genetic testing should be considered** in metastatic cases, even in the absence of syndromic features.
